# Solid Forms of Bio-Based Monomer Salts for Polyamide 512 and Their Effect on Polymer Properties

**DOI:** 10.3390/polym16212953

**Published:** 2024-10-22

**Authors:** Xiaohan Zhang, Xincao Fang, Yongliang Yan, Zihan Li, Qingshi Wen, Keke Zhang, Ming Li, Jinglan Wu, Pengpeng Yang, Junzhi Wang

**Affiliations:** 1 National Engineering Technique Research Center for Biotechnology, State Key Laboratory of Materials-Oriented Chemical Engineering, College of Biotechnology and Pharmaceutical Engineering, Nanjing Tech University, No. 30, Puzhu South Road, Nanjing 211816, China; zhang_xh31@163.com (X.Z.); fangxc77@163.com (X.F.); 15895877898@163.com (Z.L.); wenqingshi2024@163.com (Q.W.); m.li@njtech.edu.cn (M.L.); wujinglan@njtech.edu.cn (J.W.); yang-pengpeng@njtech.edu.cn (P.Y.); 2 Raybow (Hangzhou) Pharmaceutical Co., Ltd., Hangzhou 310000, China; yongliang.yan@jiuzhoupharma.com; 3 Biology+ Joint Research Center, School of Chemical Engineering and Technology, Zhengzhou University, Zhengzhou 450001, China; zhangkeke@zzu.edu.cn

**Keywords:** bio-based nylon 512, solid forms, polymerization

## Abstract

Polyamides’ properties are greatly influenced by the polymerization process and the type of feedstock used. The solid forms of nylon salts play a significant role in determining the final characteristics of the material. This study focuses on the long-chain bio-nylon 512. Firstly, we systematically investigated the possible solid forms of the nylon 512 salt, including crystal forms and morphologies, by massive experimental screening, single-crystal X-ray diffraction, Hirshfeld surface analysis, and TG-DSC measurements. The regulation and control of the various solid forms were achieved through solid-state transformations (SSTs) and solution-mediated phase transformations (SMPTs). Our findings shows that the nylon 512 salt exists in two crystal forms (anhydrate and dihydrate) and four morphologies (needle-like, plate-like, rod-like, and massive block crystal). Many factors will influence the formation of these solid forms, such as water activity, temperature, solvent, and ultrasonic physical fields. We can choose the right factors to regulate this as needed. On this basis, we studied the effects of different solid forms (crystal forms and morphologies) on the properties of the resulting polyamides prepared using direct solid-state polymerization (DSSP). The solid form of the salt had many effects on the polymer, including its structure, melting point, and mechanical properties. The polyamide obtained through DSSP of the anhydrate salt exhibited a higher melting point (204.22 °C) and greater elastic modulus (3.366 GPa) compared to that of the dihydrate salt, especially for the anhydrate salt of plate-like crystals.

## 1. Introduction

Bio-based materials are expected to gradually replace traditional petroleum-based materials in various applications, leading to increased sustainability and environmental benefits [1,2]. Nylon, also called polyamide (PA), contains a circulation unit of diamine and diacid, -[NHCO]-, on its molecular backbone [3]. In recent years, there has been significant market demand for new types of bio-nylon with excellent performance. Developing bio-based nylon not only reduces the use of petroleum resources in production to protect the environment, but also aligns with the concept of national sustainable development.

**Figure 1:**
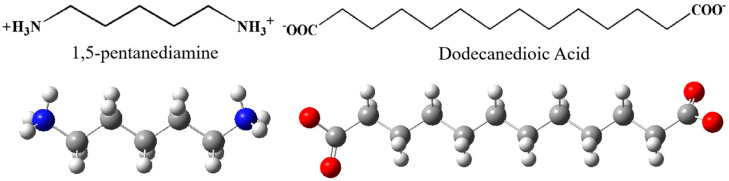
Molecular structure and chemical scheme of 1,5-pentanediamine-dodecanedioic acid.

Long-chain bio-nylon 512, polymerized by the salt 1,5-pentenediamine (PDA) [4,5] and dodecanedioic acid (DCA) [6] (Figure 1), is attracting increasing attention due to its low water absorption, strong stability, toughness, and excellent wear resistance. PDA is biosynthesized through a decarboxylation reaction driven by lysine decarboxylase from a biogenic lysine, while DCA is produced through the fermentation of mono-alkane via candida [7,8]. As a result, it is considered as a new trend, leading the future nylon material development in a new direction [9].

It is known that high-quality monomers are the foundations for high-performance polymers [10]. Currently, research on nylon polymerization mainly focuses on polymerization processes based on monomers of high chemical purity [11]. However, the solid forms of nylon monomer salts, including the crystal forms, crystal habits, particle sizes, distributions, etc., may have an impact on the polymer’s performance due to their different packing structure, symmetries, and crystallinity [12,13]. Hardness and modulus usually rise with increased crystallinity, making the product brittle. A higher crystallinity in the product leads to a more ordered chain sequence arrangement, reduced movement of molecular chain segments, lower porosity, closer interactions between molecules, and an overall improvement in density, tensile strength, thermal properties, gloss, and other polymer characteristics [14]. Thus, the first step is preparing different solid forms of the nylon 512 salt.

Effective control of the crystallization process for a nylon salt can regulate its solid form [15,16]. According to our previous contributions, six different PA5O salt monomers were discovered and obtained by the regulation of crystallization process, and four crystal forms of nylon 512 salts were also prepared by solid phase transition [17,18]. The literature has proven that water activity, temperature, humidity, and external physical fields are important factors in solid form formation [18]. Introducing a physical field during the stage of crystal nucleation or growth during solution crystallization might significantly alter the crystal form, crystal habit, and particle size distribution of nylon salt [16]. In addition, if water molecules enter the crystal lattice of nylon salts forming a crystalline hydrate, they may alter the physical properties and produce changes in thermodynamic properties with associated impacts on polymer properties [19,20].

In this paper, we investigated how to regulate the crystallization process to produce various solid forms of the PA512 salt with varying structures and morphologies. At the same time, we researched how solid forms influence polymer properties. First, we performed massive screening experiments to obtain the possible solid forms of the nylon 512 salt and determine information on its single-crystal structures and morphologies. Then, we investigated its structural features, relative stability, and mutual transformation behaviors through a combination of experiments and computation analysis, expounding the major factors that drive phase conversion and achieve regulation and control for different solid forms. On this basis, we tried to carry out polymerization of nylon 512 by combining monomers of different solid forms with direct solid-phase polymerization. The effects of different polymerization methods, polymerization temperatures, crystal forms, particle sizes, and morphologies on polymer properties were investigated.

## 2. Experimental Section

### 2.1. Materials

1,5-pentanediamine (PDA, 99.5% purity, M.P. 180 °C, M.W. 102.18 g/mol) was prepared by L-lysine decarboxylation in our laboratory; its purity and concentration were determined by high-performance liquid chromatography (HPLC). Dodecanedioic acid (99% purity, M.P. 125 °C, M.W. 230.3 g/mol) was purchased from Shanghai Macklin Biochemical Co., Ltd. (Shanghai, China). Analytical-grade organic reagents were all obtained from Shanghai Chemistry Reagent Co. (Shanghai, China). (The method of preparing this acid is the fermentation of fatty acids extracted from vegetable oils.) Deionized water was obtained from an ultrapure water system (YPYD Co., Shanghai, China).

### 2.2. Preparation of Different Crystal Forms of PA512 Salt

Anhydrate: The anhydrate of PDA-DCA was prepared by antisolvent crystallization with ethanol as solvent and ethyl acetate as antisolvent. Approximately 3.6 ± 0.1 g PDA was dissolved in 25 mL of ethanol at room temperature in a 250 mL double-jacket crystallizer. A three-bladed paddle (HD2015W, Thra instruments, Shanghai, China) stirred the solution at 200 r/min, and 8.2 g of DCA powder was added slowly. After the solution became completely transparent, the mixture was kept stirring for 30 min. We pumped ethyl acetate into the crystallizer at 0.4 mL/min with a peristaltic pump. When the accumulative volume of the antisolvent approached about 25 mL, the cloud point occurred. The antisolvent addition continued until its total feeding volume was 10 times that of the ethanol. The system was stirred for an additional 2 h, resulting in the formation of anhydrate.

Dihydrate: The PDA-DCA dihydrate was prepared by cooling crystallization; 7.2 g of PDA and 16.4 g of DCA were dissolved in 35 mL of water at 55 °C and then transferred to a 100 mL crystallizer. The solution was cooled at a rate of 10 °C/min after maintaining equilibrium for 30 min, initiating crystallization around 15 °C. We kept the system stirring for 2 h to obtain the dihydrate of PDA-DCA.

### 2.3. Single-Crystal X-Ray Diffraction (SCXRD) and Powder X-Ray Diffraction (PXRD)

Single-crystal X-ray diffraction (SCXRD) of different crystal forms was measured at 293 K using a Bruker SMART APEX (Bruker, Germany) diffractometer (λ = 0.71073 Å) equipped with Mo Kα radiation. Data were collected—taking advantage of the Bruker SMART-1000 project (Bruker, Germany)—as well as being indexed, integrated, scaled using APEX3, and reduced utilizing the SAINT program [21]. All non-hydrogen atoms were anisotropic and refined. Hydrogen atoms are fixed in ideal molecular geometry and assigned isotropic thermal parameters according to the equivalent displacement parameters of the atoms to which they are bound. Detailed crystallographic data for refinement parameters and all crystal structures were processed with PLATON [22]. Structural visualization and analysis were performed via Olex2 1.3.0 [23] and Mercury 2020.2.0 [24].

Powder X-Ray diffraction (PXRD) of solid powders was carried out by Cu Kα radiation (λ = 1.5406 Å) (Rigaku SmartLab, Tokyo, Japan). We, respectively, set the intensity and voltage of the X-ray source to 40 mA and 45 kV. The sample was scanned in transmission mode at a rate of 10° min^−1^ with a step size of 0.02° in the range of 5° to 45°.

### 2.4. Phase Transformation Behaviors of Different Crystal Forms

Effect of temperature: Approximately 5 g of solid powder was placed in the Petri dish and subjected to various temperature conditions (40, 60, 80, 90, 110 °C) for 4–6 h. The solid structure of the samples was monitored using PXRD.

Effect of water activity: Within the experimental temperature range of 298.15–323.15 K, excessive mixtures of two crystalline powders at a mass ratio of 1:1 were stirred for 48 h in a series of water–ethanol binary solvents with various water activity, until the phase equilibrium was achieved. Table 1 presents the amount of water added to the ethanol during the experiments. The slurry was filtered, and the wet cake was taken out, dried, and ground. The resulting solid samples were identified by PXRD. The crystals were observed using a polarizing microscope (MP41, Guangzhou Mingmei Photoelectric Technology Co., Ltd., Guangzhou, China).

### 2.5. Fourier Transform Infrared Spectroscopy with Attenuated Total Reflectance (ATR-FTIR)

Infrared spectra were measured using ATR total reflection with an FTIR (NICOLET iS5, Thermo Fisher Scientific, Waltham, MA, USA) spectrometer. The measurement range was 4000 to 600 cm^−1^ with a resolution of 4.0 cm^−1^, and 256 scans per spectrum were conducted under ambient conditions, using atmospheric data for background correction.

### 2.6. Thermogravimetry and Differential Scanning Calorimetry (TGA-DSC)

TGA-DSC measurements (NETZSCH STA 449 F3, NETZSCH, Selb, Germany) were conducted to characterize the thermal behavior of PA512 salt of different crystal forms. A sample of 2–5 mg was placed in an alumina crucible and heated from 40 °C to 400 °C at a rate of 10 °C/min in nitrogen atmosphere, while thermogravimetric and thermal effect curves were recorded. As for the polymer, DSC measurements were performed using a TA Q20, with a nitrogen flow rate of 40 mL/min. The temperature of the crucible containing 3–5 mg of the sample was raised to 300 °C at a rate of 10 °C/min and held for 1 min to eliminate thermal history. The sample was then cooled to 0 °C at 10 °C/min, held at that temperature for 1 min, and subsequently heated back to 300 °C at the same rate, allowing for the tracking of DSC curves.

### 2.7. Hirshfeld Surface Analysis (HSs)

Crystal Explorer 21.5 software was utilized to analyze the molecular Hirshfeld surfaces (HSs) [25] and related 2D fingerprint patterns. Specifically, this software enables the exploration of intermolecular interactions, quantification of different contact types, and comparison of lattice energies [26,27].

### 2.8. Polymerization

Direct solid-state polymerization (DSSP): PA512 salt powder was placed in an autoclave, and the program was divided into four steps:

1. Nitrogen was purged into the autoclave multiple times to replace residual air. The autoclave reactor was then heated to 110~120 °C, maintaining a pressure of 0.5 MPa for pre-polymerization for 1 h.
2. The polymerization temperature was increased to 140 °C at a rate of 0.2 °C/min for 1 h.
3. We reduced the pressure in the autoclave to atmospheric pressure within 1 h.
4. At last, the reaction was carried out under complete vacuum conditions for 0.5 h. Finally, a light, white, plastic product was obtained at the bottom of the reaction kettle.

Melting polymerization (MP): The solid powder of PDA-DCA 5 g and 10 mL of water were added to the 100 mL autoclave reactor, and N_2_ was used to purge the air from the reactor. The reaction temperature was raised to 120 °C for pre-polymerization, which was conducted for 1 h. The temperature was then increased to 140 °C, and the polymerization reaction was carried out for 1 h. The pressure was slowly reduced to normal pressure over 1 h, and the reaction continued for an additional 0.5 h under complete vacuum conditions. After cooling to room temperature and atmospheric pressure, the sample was removed from the reactor.

### 2.9. Nanoindentation Technology

Resin was used for inlay sample preparation. An indenter with a hardness greater than that of the sample was employed to measure the hardness and elastic modulus of the material by continuously recording the test force and indentation depth. The testing instrument was the Nanotest Alpha (Micro Materials Ltd., Wrexham, UK). The test parameters are 10 mN load, 30 s load time, and 5 s load retention.

## 3. Results and Discussion

### 3.1. Structure Analysis of Different Solid Forms for PA512 Salt

#### 3.1.1. Single-Crystal X-Ray Diffraction

We found and obtained two crystal forms (dihydrate and anhydrate) of PDA-DCA. Their specific crystallographic information was first analyzed by SCXRD, listed in Table 2, and has been deposited in the Cambridge Crystallographic Data Centre. Both forms belong to the triclinic crystal system with a *P*-1 space group. Notably, the dihydrate contains two water molecules in its crystal lattice, whereas the anhydrate does not. Additionally, the crystal densities differ: the anhydrate has a density of 1.090 g/cm^3^, while the dihydrate has a density of 1.137 g/cm^3^.

The solid samples used in this study were all characterized by PXRD, shown in Figure S1. It can be found that the experimental patterns were in good agreement with the simulation pattern from their respective single crystals. The characteristic peaks of anhydrate were located at 9.30°, 13.98°, 19.30°, 21.34°, and 24.88° (2θ), while the dihydrate exhibited peaks at 5.86°, 11.34°, 19.80°, 20.82°, 21.02°, 21.40°, 22.76°, and 24.26° (2θ), which can easily be used to distinguish between the two crystal forms. Infrared spectroscopy mainly provides abundant information about molecular functional groups and chemical bonds. FTIR spectrogram analysis can also serve as a tool for analyzing and identifying the two different crystal forms, as displayed in Figure S2. Additionally, TGA and DSC were employed to analyze the thermal behavior of the crystals, as depicted in Figure S3.

#### 3.1.2. Structural Features of Two Crystal Forms

**Figure 2:**
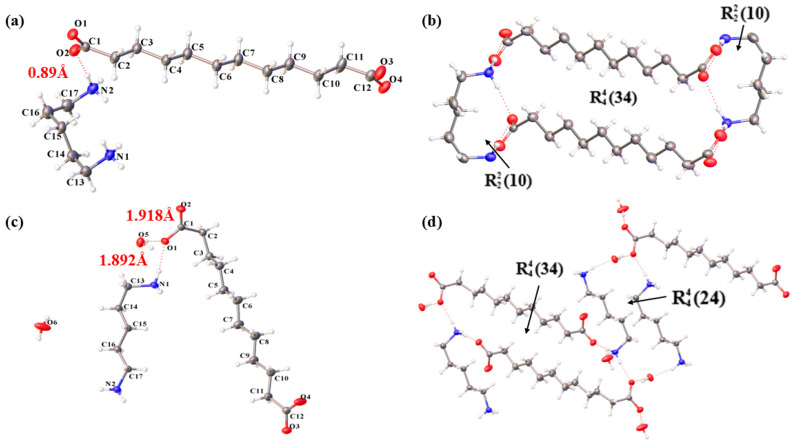
(**a**) Asymmetric unit of PDA-DCA anhydrate, with a 30% probability of ellipsoidal display and (**b**) its 2D structure. (**c**) Asymmetric unit of dihydrate, with a 30% probability of ellipsoidal display and (**d**) its 2D structure.

Figure 2 illustrates the asymmetric unit diagrams for the two crystal forms of PDA-DCA. Their differences were mainly embodied in the different supramolecular synthons, molecular stacking modes, and conformation. When the water molecule was introduced to the crystal lattice, the interaction force between binary amine and binary acid became complicated and changed [28] and the PDA molecule reversed substantially [29], as depicted in Figure 2a,d, specifically reflected in anhydrate (φ_1_(N_1_-C_13_-C_14_-C_15_) = 57.8°, φ_2_(C_14_-C_15_-C_16_-C_17_) = −64°, and φ_3_(C_15_-C_16_-C_17_-N_2_) = 55.4°) and dihydrate: φ_1_(N_1_-C_13_-C_14_-C_15_) = 27.24°, φ_2_(C_14_-C_15_-C_16_-C_17_) = 177.25°, and φ_3_(C_15_-C_16_-C_17_-N_2_) = 176°. As water molecules enter the crystal lattice, the amidogen of PDA molecules in the dihydrate make an adjustment and shift to better adapt to interactions with water and DCA molecules. Compared with dihydrate, the torsion of PDA in the anhydrous crystal was more obvious. The amino groups at both ends of the PDA molecule twist to the same side, and the dihedral angle φ_3_ twists almost into a plane [30].

Figure 2a reveals that anhydrate of PDA-DCA only has one N-H…O strong hydrogen bonding interaction [31]. The amino groups at both ends of PDA connect with the carboxyl groups of adjacent DCA to form a closed-loop synthon of $R_{2}^{2}$(10) [32,33], while the four carboxyl groups at both ends of adjacent dodecanedioic acid connect with the two amino groups at both ends of PDA to form a synthon of $R_{4}^{4}$(34), as depicted in Figure 2b. From Figure 2c, compared with anhydrate, there are two kinds of hydrogen bonds in the crystal structure of the dihydrate that contact N-H…O and O-H…O; H atoms on water molecules and O atoms on the carboxyl group of DCA interact through a hydrogen bond. From Figure 2d, the dihydrate not only forms a synthon $R_{4}^{4}$(34) similar to the anhydrate, but also, the adjacent PDA is linked to the carboxyl group on the DCA molecule through water molecules to form a new supramolecular synthon $R_{4}^{4}$(24).

#### 3.1.3. Molecular Interaction Analysis in Crystal Lattice of Two Crystal Forms

**Figure 3:**
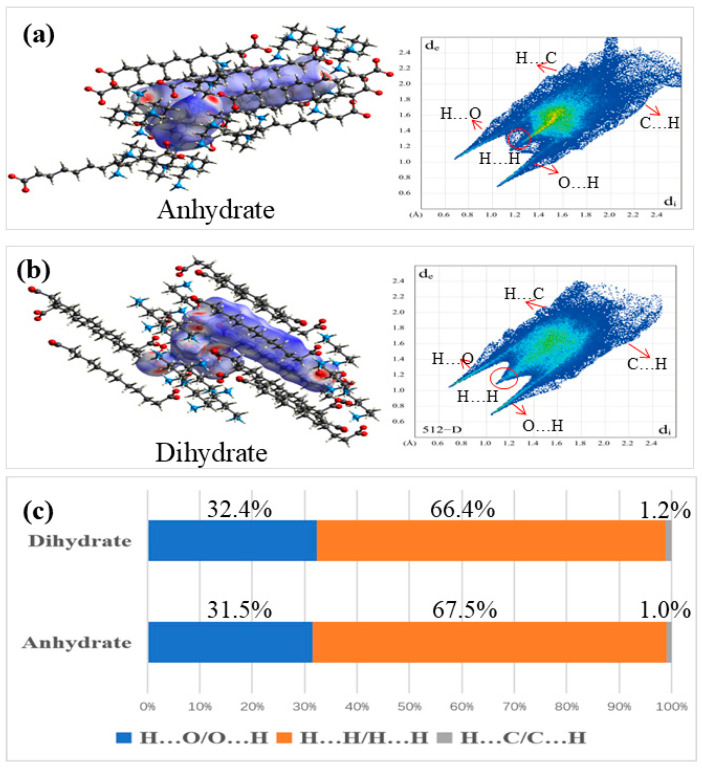
Hirshfeld surface analysis and fingerprint analysis of two crystal forms of PDA-DCA: (**a**) anhydrate, (**b**) dihydrate, and (**c**) statistical analysis.

We used Hirschfeld surface analysis to describe the interactions between molecules more clearly and quantitatively [34]. The 2D fingerprints can quantify the level of contact interactions between molecules by highlighting specific close contacts. The Hirshfeld surface analysis was performed and 2D fingerprint patterns of PDA-DCA for two crystal forms are displayed in Figure 3.

According to the Hirshfeld surface analysis map, when the intermolecular interactions are strong, bright red dots will be displayed, indicating that hydrogen bonds such as O-H∙∙O are in closer contact, while weak red dots represent some weak hydrogen bonds. The 2D fingerprints visualize the interactions and quantitative analysis. PDA-DCA has a relatively simple interaction force, including three kinds of interaction forces: H∙∙O\O∙∙H, H∙∙C\C∙∙H, and H∙∙H\H∙∙H. It was noteworthy that the O··H/H··O contacts were visible as a pair of symmetrical ‘winglike’ spikes, representing hydrogen bonding ratios of 31.5 (anhydrate) and 32.4 (dihydrate). The H··H interface, which looks like the “head” in the middle of the wing, has the highest proportion, indicating that both crystal forms are stable. The remaining relatively weak interactions occur on the wing sides.

### 3.2. Phase Transition, Regulation, and Control of Two Crystal Forms for PA512 Salt

We tried to investigate the mutual transformation relationship of anhydrate and dihydrate of PA512 salt, elucidating the major factor to drive phase conversion, to develop a regulation and control strategy for different crystal forms.

#### 3.2.1. Solid-State Transformation (SST)

**Figure 4:**
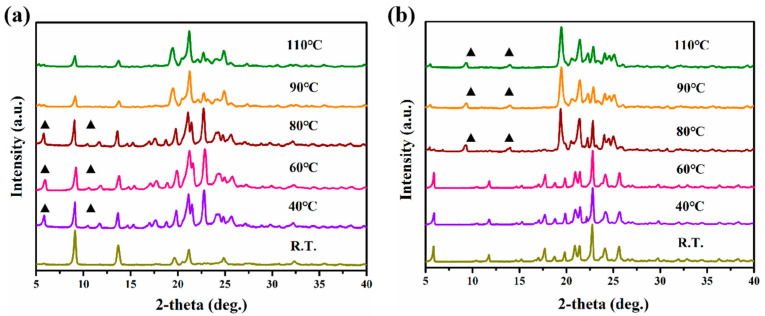
The SST process of the anhydrate (**a**) and dihydrate (**b**) under different temperatures.

Solid-state transformation is a common method for studying phase transition, as verified by PXRD [35]. Figure 4a reveals that the anhydrate begins to transform into dihydrate at 40 °C, and mixed crystal appears. As the temperature rises to 90 °C, the water molecules are completely lost from the lattice; then, the hydrate changes into anhydrate again. For the dihydrate, dehydration occurs at 80 °C, fully transforming into anhydrate after 30 min, as illustrated in Figure 4b.

#### 3.2.2. Solution-Mediated Phase Transformation (SMPT)

**Figure 5:**
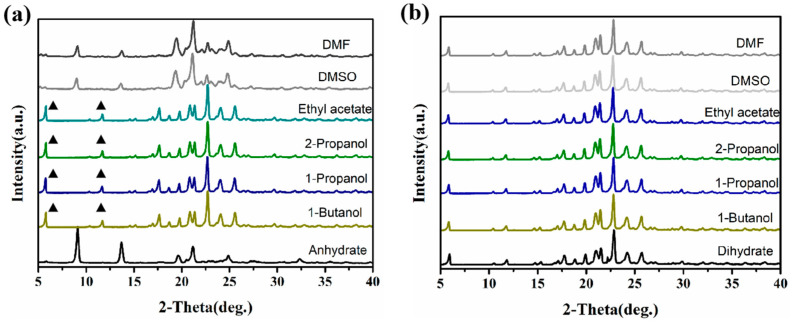
SMPT diagrams of two crystal forms of PA512 salt: (**a**) anhydrate and (**b**) dihydrate.

We conducted SMPT experiments using different solvents at room temperature to investigate their influence on crystal form transitions and explore the possible occurrence of new crystal forms [36]. As shown in Figure 5, no new phases were observed. We discovered that the anhydrate transformed into dihydrate in ethyl acetate, 1-propanol, 2-propanol, or 1-butanol, while the dihydrate maintained its original crystal structure. This suggests that the dihydrate may exhibit greater stability compared to its anhydrate counterpart.

#### 3.2.3. Effect of Water Activity on Crystal Forms

**Figure 6:**
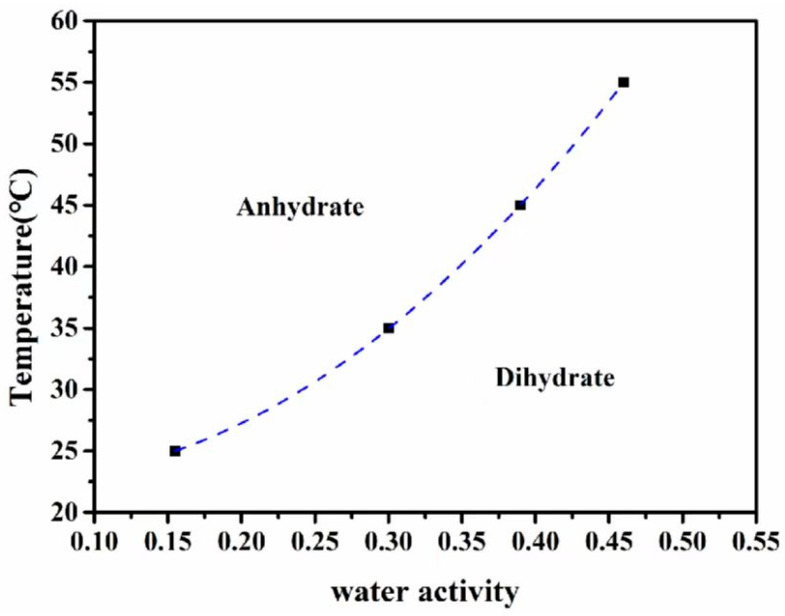
Temperature–water activity phase diagram of two crystal forms of PA512 salt.

During the experiment, we primarily obtained the dihydrate form of the crystals, while the anhydrate form was more challenging to obtain. Previous research has indicated that water activity is a crucial factor in controlling crystal form [37]. To investigate this, we conducted a slurry experiment by adding an excess mixture powder with a mass ratio of 1:1 of both crystal forms to a reaction flask and performed SMPT in various proportions of binary solvents at different temperatures; the temperature and the ratio of water to ethanol are shown in Table 2. As shown in Figure 6, at room temperature, a water activity of no more than 0.15 was sufficient for easy transformation from anhydrate to dihydrate, indicating that the feasible range of obtaining anhydrate was relatively narrow. Additionally, the critical value of required water activity increased with the conversion temperature; the higher the conversion temperature, the greater the required water activity. The phase diagram in Figure 6 clearly defines the transition boundary between the hydrate zone and the anhydrate zone.

**Figure 7:**
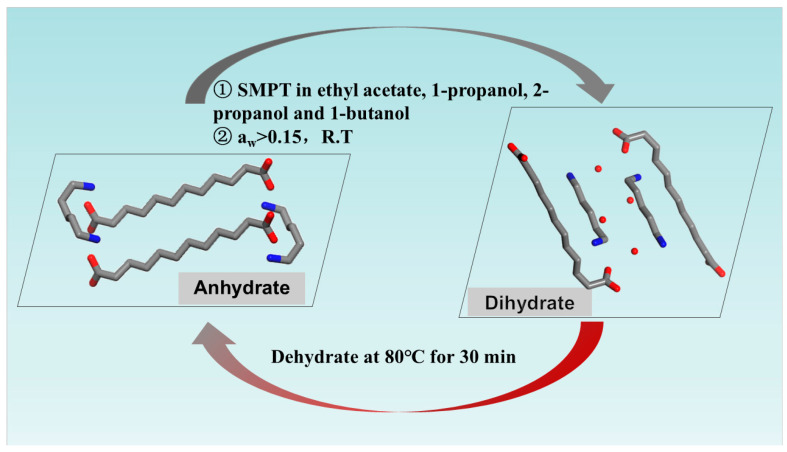
Regulation and control of two crystal forms of PA512 salt.

In conclusion, Figure 7 illustrates the mutual transformation relationship between the anhydrate and dihydrate forms of PA512 salt. The anhydrate can be converted to the dihydrate via SMPT in solvents such as ethyl acetate, 1-propanol, 2-propanol, or 1-butanol for 12 h, or by translation for 12 h within a water activity of 0.15 (water–ethanol) at room temperature. Similarly, the dihydrate can be transformed into the anhydrate through dehydration in a vacuum drying oven at 80 °C for 30 min.

### 3.3. Morphology Regulation of PA512 Salt

**Figure 8:**
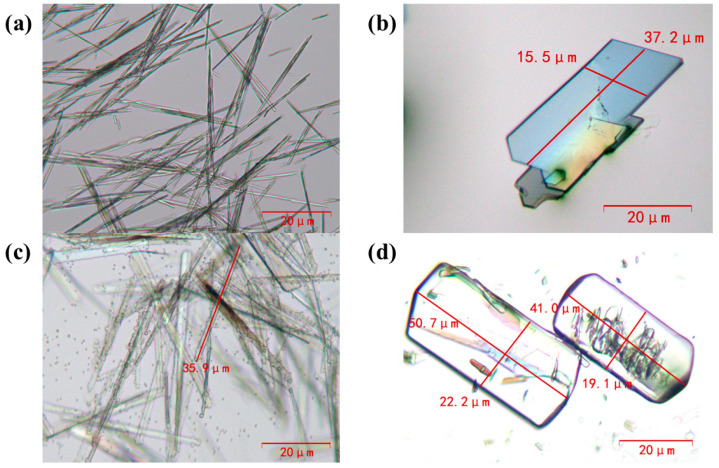
Polarized light micrographs of the PDA-DCA salt: (**a**) anhydrate; (**b**) anhydrate with ultrasound (100 w^−1^ h); (**c**) dihydrate; (**d**) dihydrate with ultrasound (100 w^−1^ h). The magnifications are 10 × 4.

Figure 8a,c illustrate the different crystal habits of samples with different crystal forms. The anhydrate is needle-like while the dihydrate is rod-like. Ultrasound can break the crystals to a certain extent, controlling their size, and the resulting cavitation bubbles can significantly affect crystal growth [38]. When combined with crystallization processes, ultrasonic action can effectively improve crystal habits. As shown in Figure 8b, through the coupling of dissolution crystallization and ultrasonic pretreatment with a 100 W probe for 60 min, the morphology of anhydrate changed from needle crystals to quadrilateral sheets. For the preparation of the dihydrate, water bath ultrasonic pretreatment (100 W, 60 min) was used before cooling crystallization, which transformed the dihydrate from rod-like to massive, as illustrated in Figure 8d.

### 3.4. Polymerization Analysis

#### 3.4.1. Effect of Polymerization Temperature

**Figure 9:**
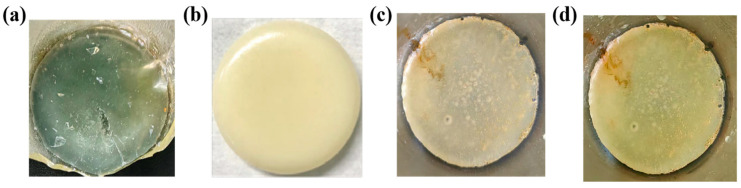
Diagrams of PA512 at various temperatures: (**a**) 130 °C; (**b**) 140 °C; (**c**) 150 °C; (**d**) 160 °C.

**Figure 10:**
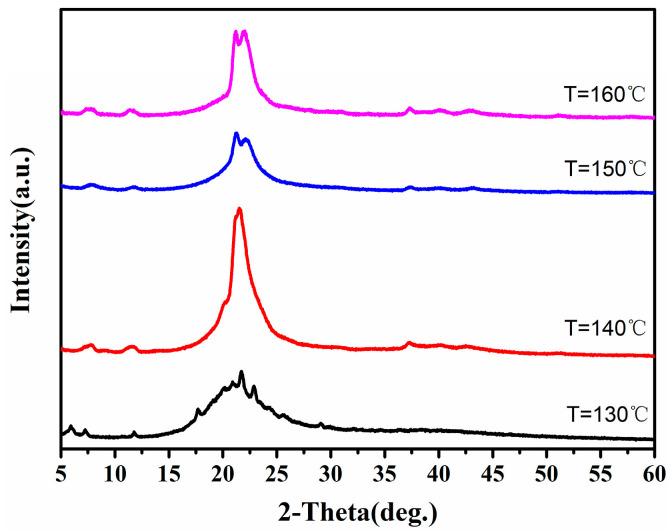
PXRD of PA512 at different temperatures.

We optimized the effect of different polymerization temperatures on product quality under direct solid-state polymerization (DSSP) using the anhydrate of PA512 salt, as illustrated in Figure 9. At a polymerization temperature of 140 °C, the polymer exhibited the lightest and smoothest appearance, with a glossy finish. The polymer’s PXRD and TGA-DSC results are presented in Figure 10 and Table 3, respectively. When the polymerization temperature was set at 140 °C, the resulting product exhibited the highest crystallinity and better thermal stability. Therefore, to ensure optimal product performance, the polymerization temperature of 140 °C was selected.

#### 3.4.2. Effect of Polymerization Method and Crystal Forms on Polymer Properties

**Figure 11:**
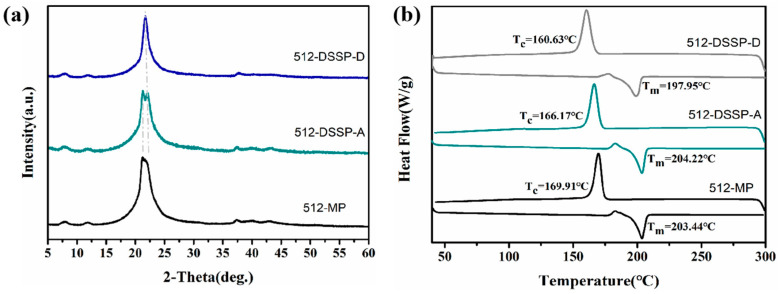
PXRD (**a**) and DSC (**b**) characterization of different PA512 products. 512-MP represents melting polymerization with anhydrate as raw material. 512-DSSP-A and 512-DSSP-D stand for solid-phase polymerization with anhydrate and dihydrate as raw material, respectively.

The effects of different crystal forms (anhydrate and dihydrate) of PA512 salt on the polymerized product were investigated using DSSP at a polymerization temperature of 140 °C, and traditional melting polymerization (MP) was also performed as a contrast, as illustrated in Figure 11a. Notably, structural differences between the products were obtained from different crystal forms. The characteristic peak of the polymerized product obtained from anhydrate as raw material exhibits an obvious double peak around 21.5° of 2θ in the PXRD pattern, while that of the dihydrate as raw material displays a single sharp peak.

Table 4 presents the results of molecular weight determination of nylon obtained through different polymerization methods and with different crystal forms using gel permeation chromatography (GPC), showing that the molecular weight of the PA512 product obtained by DSSP is more than 35 KDa, which is higher than MP and indicates excellent polymerization efficiency. The thermal behavior analysis in Figure 11b reveals that the melting points of 512-DSSP-A and 512-DSSP-D are notably different at values of 204.22 °C and 197.95 °C. The recrystallization temperature of 512-DSSP-A is 166.17 °C, which is nearly 6 °C higher than that of 512-DSSP-D (160.63 °C). The hardness of PA512-DSSP-D is similar to that of 512-DSSP-A, but the polymerization product obtained from the anhydrate crystal form of PA512 salt has a higher elastic modulus with 3.366 GPa. These findings suggest that the choice of raw material crystal form significantly affects the polymerization result and properties of the resulting polymer.

#### 3.4.3. Effect of Particle Morphology of PA512 Salt on Polymer Properties

**Figure 12:**
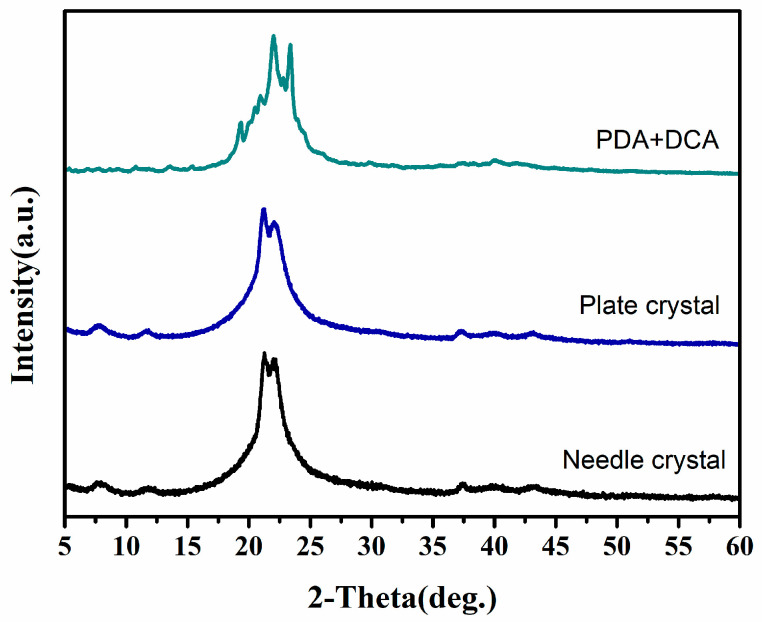
PXRD characterization of polymerized products with different raw materials under DSSP optimization conditions.

**Figure 13:**
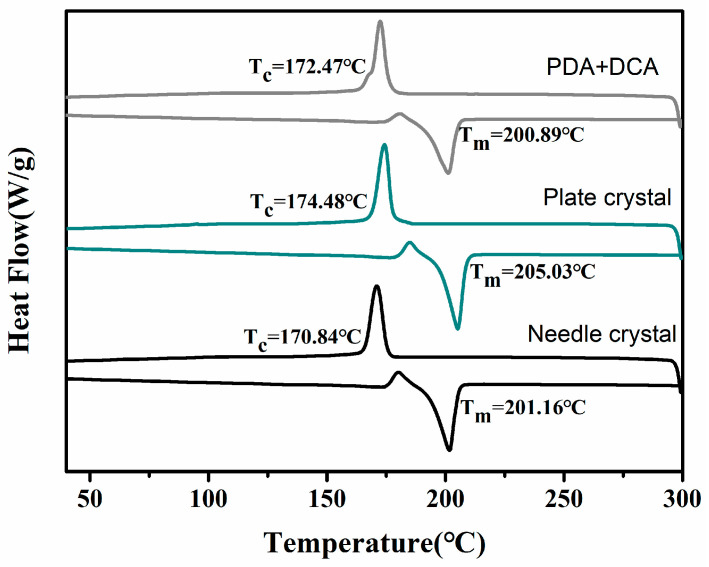
DSC of polymerized products with different raw materials under DSSP optimization conditions.

In the case of DSSP, factors such as crystal size, size distribution and crystal habit (fiber, needle, block, etc.) may influence the polymerization process and polydispersity of the polymer by altering the number of repeating units. In this study, we investigated two types of PA512 salt with different particle morphologies as raw materials for polymerization. To eliminate the influence of crystal water molecules, the above-mentioned anhydrate was used as the feedstock. One is the original needle crystalline powder; the other is plate crystals obtained by ultrasonic treatment, and the direct dehydrated PDA and DCA were used as a contrast. The results are shown in Figure 12. As shown in Figure 13, The polymerized products obtained by PA512 crystalline anhydrate salt appear to have a higher melting point, and that of massive anhydrate crystals exhibits greater hardness and elastic modulus with 3.7 GPa (Table 5).

The relationship between relative crystallinity *X_T_* and crystallization temperature *T* is shown in Equation (1):

(1) $$X_{T}=\frac{\int_{T_{0}}^{T}\frac{dH_{c}\left(T\right)}{d\left(T\right)}}{\int_{T_{0}}^{T_{\infty }}\frac{dH_{c}\left(T\right)}{d\left(T\right)}}$$

The relative crystallinity (*X_T_*) of the product was calculated based on the ratio of the measured melting enthalpy to the theoretical enthalpy of 100% crystalline material, as shown in Table 5. The hardness and elastic modulus of the polymer are enhanced with crystallinity, thereby, making the product hard and brittle. The higher the crystallinity of the product, the more orderly the chain sequence arrangement, the closer the interaction between molecules, the more difficult the movement of molecular chain segments, and the lower the porosity. When using plate crystals as polymerization material, the product has higher crystallinity and better performance.

## 4. Conclusions

In this study, we investigated the solid forms of the long-chain bio-based polyamide 512 salt and their effects on polymer properties. We found and prepared PA512 salt with different crystal forms (anhydrate and dihydrate) and different crystal morphologies (needle, plate, massive block, particle size). Then, we systematically analyzed the structural features, intermolecular action in crystal lattices, relative stability, and phase transition behaviors of two crystal forms of PA512 salt by single-crystal X-ray diffraction, Hirshfeld surface analysis, TG-DSC, solid-state transformation (SST), and solution-mediated phase transition (SMPT). There are significant differences in single PDA and TCA molecular conformation in two lattices, as well as packing arrangements for two crystal forms. Our findings indicated that the stability of the dihydrate is relatively more favorable than that of the anhydrate. The preparation of two crystal forms can be regulated and controlled by water activity, temperature, and solvent. In addition, the crystal habit and particle size of two crystal forms of PA512 salt were dependent on their respective microscopic molecular arrangement as well as ultrasonic stimulation during crystallization. The needle-like anhydrate and rod-like dihydrate can be transformed into plate or massive block crystals by ultrasonic auxiliary treatment for 60 min, respectively.

Based on this, we investigated the effects of different crystal forms and morphologies of PA512 salt on their polymerized product properties by direct solid-state polymerization (DSSP). The structures of the resulting polymers were obviously influenced by the crystal forms of raw materials. The polymerized product of anhydrate by DSSP possesses a unique melting point (204.22 °C) and greater elastic modulus (3.366 GPa). Furthermore, the polymer based on the plate anhydrate crystals of PA512 salt demonstrated greater hardness (0.231 GPa) and elastic modulus (3.7 GPa), while those of the needle-like crystal were 0.211 GPa and 3.366 GPa, respectively. The solid form of PA512 polymerization feedstock—referring to both crystal form and morphology—affected the structure, melting point, and mechanical properties of the resulting polymer. In this paper, we improve the performance of the polymer by regulating the solid forms of the polymerization monomer, providing a reference for performance regulation of other polyamides.

## Figures and Tables

**Table 1:** Percentage of water in the water–ethanol system.

Temperature/°C	Water Content/%									
25	0	2	4	6	8	10	12	14	16	18
35	11	12	13	14						
45	11	12	13	14						
55	15	16	17	18	19					

**Table 2:** Crystallographic data for PDA-DCA anhydrate and dihydrate.

	Anhydrate	Dihydrate
**Empirical formula**	C_12_ H_20_ O_4_, C_5_ H_16_ N_2_	C_12_ H_20_ O_4_, C_5_ H_16_ N_2_, 2(H_2_O)
**Formula weight**	332.48	368.51
**Crystal system**	triclinic	triclinic
**Space group**	*P*-1	*P*-1
**Temperature(K)**	298	298
***a* (Å)**	5.7474(7)	8.4747(7)
***b* (Å)**	9.5278(9)	8.5175(7)
***c* (Å)**	19.1712(18)	15.3901(14)
***α* (°)**	82.258(2)	82.473(2)
***β* (°)**	89.152(3)	78.060(1)
***γ* (°)**	76.817(1)	86.313(2)
**Volume (Å^3^)**	1012.70(18)	1076.71(16)
***Z***	2	2
**D_calc._(g cm^−3^)**	1.090	1.137
***μ* (mm^−1^)**	0.076	0.084
***F* (000)**	368	408
**Crystal size (mm)**	0.46 × 0.30 × 0.15	0.49 × 0.40 × 0.20
**R(int)**	0.042	0.022
**R1[I > 2σ(I)]**	0.1270	0.0532
**wR^2^**	0.3020	0.1438
**GOF on F^2^**	1.198	1.062
**Diff. density (e Å^−3^)**	0.736, −0.317	0.253, −0.295
**CCDC**	2156810	1917983

**Table 3:** The melt point (T_m_) and crystallization temperature (T_c_) of PA512 at various temperatures.

Polymerization Temperature (°C)	T_m_ (°C)	T_c_ (°C)
130	200.33	163.67
140	207.47	169.10
150	206.34	169.06
160	210.11	170.19

**Table 4:** Comparison of characteristics of the three kinds of polymers obtained.

	*M*_n_/Da	*M*_w_/Da	PDI	T_melt_/°C	Hardness (GPa)	Elastic Modulus (GPa)
MP	13,828	26,175	1.89	203.44	0.211	3.019
DSSP-A	18,336	35,601	1.94	204.22	0.219	3.366
DSSP-D	23,772	41,175	1.73	197.95	0.218	3.038

**Table 5:** Comparation of the molecular weight and thermodynamic properties of PA 512 with different raw materials.

	*M*_n_/Da	*M*_w_/Da	PDI	Hardness (GPa)	Elastic Modulus (GPa)	Xc (%)
PDA+DCA	14,816	38,618	2.61	0.211	3.611	50.64
Needle crystal	18,336	35,601	1.94	0.213	3.399	44.94
Plate crystal	15,754	39,086	2.48	0.231	3.700	56.08

## Data Availability

Data are contained within the article and Supplementary Materials.

## Supplementary Materials

The following supporting information can be downloaded at https://www.mdpi.com/article/10.3390/polym16212953/s1: Figure S1: Comparison of powder X-ray diffraction (PXRD) patterns with the simulated patterns from single-crystal X-ray diffraction (SCXRD) for the anhydrate and dihydrate of PDA-DCA; Figure S2: Comparison of the FTIR patterns of the nylon 512 salt anhydrate and dihydrate; Figure S3: DSC and TGA curves of anhydrate (a) and dihydrate (b).

## References

[1] Kalaj M., Denny Jr M.S., Bentz K.C., Palomba J.M., Cohen S.M. (2019). Nylon–MOF Composites through Postsynthetic Polymerization. Angew. Chem..

[2] Gu J., Lv Z., Wu Y., Guo Y., Tian L., Qiu H., Li W., Zhang Q. (2017). Dielectric thermally conductive boron nitride/polyimide composites with outstanding thermal stabilities via in-situ polymerization-electrospinning-hot press method. Compos. Part A Appl. Sci. Manuf..

[3] Park S., Park H.H., Ko Y.-S., Lee S.J., Le T.S., Woo K., Ko G. (2017). Disinfection of various bacterial pathogens using novel silver nanoparticle-decorated magnetic hybrid colloids. Sci. Total Environ..

[4] Kind S., Wittmann C. (2011). Bio-based production of the platform chemical 1,5-diaminopentane. Appl. Microbiol. Biotechnol..

[5] Ikeda N., Miyamoto M., Adachi N., Nakano M., Tanaka T., Kondo A. (2013). Direct cadaverine production from cellobiose using β-glucosidase displaying *Escherichia coli*. AMB Express.

[6] Funk I., Rimmel N., Schorsch C., Sieber V., Schmid J. (2017). Production of dodecanedioic acid via biotransformation of low cost plant-oil derivatives using Candida tropicalis. J. Ind. Microbiol. Biotechnol..

[7] Matsushima Y., Hirasawa T., Shimizu H. (2016). Enhancement of 1,5-diaminopentane production in a recombinant strain of Corynebacterium glutamicum by Tween 40 addition. J. Gen. Appl. Microbiol..

[8] Ayorinde F.O., Powers F.T., Streete L.D., Shepard R.L., Tabi D.N. (1989). Synthesis of dodecanedioic acid *fromvernonia galamensis* oil. J. Am. Oil Chem. Soc..

[9] Ma W., Cao W., Zhang B., Chen K., Liu Q., Li Y., Ouyang P. (2015). Engineering a pyridoxal 5′-phosphate supply for cadaverine production by using *Escherichia coli* whole-cell biocatalysis. Sci. Rep..

[10] Othman N.S.e., Févotte G., McKenna T.F. (2004). Biobjective control of emulsion polymerizations: Control of the polymer composition and the concentration of monomer in the polymer particles. Chem. Eng. J..

[11] Ding Y., Liu C., Zhou X., Wang Z., He J., Jiang F., Wang Z. (2023). Eco-plastics derived from low-purity plant oil monomer and their sustainable recycling. Cell Rep. Phys. Sci..

[12] Yang X., Zhong Z., Xu J., Huang Y. (2017). Drug-polymer inclusion complex as a new pharmaceutical solid form. Chin. Chem. Lett..

[13] Li Z., Li S., Yang P., Fang X., Wen Q., Li M., Zhuang W., Wu J., Ying H., MacGillivray L.R. (2023). The effect of polymorphism on polymer properties: Crystal structure, stability and polymerization of the short-chain bio-based nylon 52 monomer 1,5-pentanedi amine oxalate. IUCrJ.

[14] Wu T.M., Blackwell J.J.M. (1996). Comparison of the Axial Correlation Lengths and Paracrystalline Distortion for Technora and Kevlar Aromatic Polyamide Fibers. Macromolecules.

[15] Li Z., Xu M., Liu H., Wen Q., Fu J., Zhuang W., Yang P., Wu J., Ying H. (2020). Monohydrate and anhydrate of nylon 5I monomer 1,5-pentanediamine–isophthalate. RSC Adv..

[16] Morosini V., Chave T., Virot M., Moisy P., Nikitenko S.I. (2016). Sonochemical water splitting in the presence of powdered metal oxides. Ultrason. Sonochem..

[17] Otsuka M., Ishii M., Matsuda Y. (2003). Effect of surface modification on hydration kinetics of carbamazepine anhydrate using isothermal microcalorimetry. AAPS PharmSciTech.

[18] Allan M., Chamberlain M.C., Mauer L.J. (2019). RH-Temperature Stability Diagram of the Dihydrate, β-Anhydrate, and α-Anhydrate Forms of Crystalline Trehalose. J. Food Sci..

[19] Betigeri S., Thakur A., Shukla R., Raghavan K. (2008). Effect of polymer additives on the transformation of BMS-566394 anhydrate to the dihydrate form. Pharm. Res..

[20] Hedicke K., Wittich H., Mehler C., Gruber F., Altstädt V. (2006). Crystallisation behaviour of Polyamide-6 and Polyamide-66 nanocomposites. Compos. Sci. Technol..

[21] Sheldrick G.M. (2015). Crystal structure refinement with SHELXL. Acta Crystallogr. Sect. C Struct. Chem..

[22] Spek A. (2003). Single-crystal structure validation with the program PLATON. J. Appl. Crystallogr..

[23] Dolomanov O.V., Bourhis L.J., Gildea R.J., Howard J.A.K., Puschmann H. (2009). OLEX2: A complete structure solution, refinement and analysis program. J. Appl. Crystallogr..

[24] Gavezzotti A., Filippini G., Tsoucaris G. (1999). Self-Organization in Molecular Crystals, Liquids and Solutions: Computer Studies. Current Challenges on Large Supramolecular Assemblies.

[25] Hirshfeld F.L. (1977). Bonded-atom fragments for describing molecular charge densities. Theor. Chim. Acta.

[26] Spackman P.R., Turner M.J., McKinnon J.J., Wolff S.K., Grimwood D.J., Jayatilaka D., Spackman M.A. (2021). CrystalExplorer: A program for Hirshfeld surface analysis, visualization and quantitative analysis of molecular crystals. J. Appl. Crystallogr..

[27] Spackman M.A., McKinnon J.J. (2002). Fingerprinting intermolecular interactions in molecular crystals. CrystEngComm.

[28] Guguta C., Meekes H., de Gelder R. (2006). The crystal structure of aspartame anhydrate from powder diffraction data. Acta Crystallogr. Sect. A.

[29] Putra O., Toyoshima R., Ibrahim S., Uekusa H. (2014). Dehydration mechanism of Ciprofloxacin Hydrochloride hydrate crystal. Acta Crystallogr. Sect. A.

[30] Li Z., Yang P., Liu H., Liu J., Zhu S., Li X., Zhuang W., Wu J., Ying H. (2020). Crystal forms and phase transformation of 1,5-pentanediamine-terephthalate: A bio-based nylon 5T monomer. Acta Crystallogr. Sect. B Struct. Sci. Cryst. Eng. Mater..

[31] Bernstein J., Davis R.E., Shimoni L., Chang N.-L. (1995). Patterns in Hydrogen Bonding: Functionality and Graph Set Analysis in Crystals. Angew. Chem. Int. Ed. Engl..

[32] Etter M.C., MacDonald J.C., Bernstein J. (1990). Graph-set analysis of hydrogen-bond patterns in organic crystals. Acta Crystallogr. Sect. B Struct. Sci..

[33] Etter M.C. (1990). Encoding and decoding hydrogen-bond patterns of organic compounds. Acc. Chem. Res..

[34] Al-Resayes S.I., Azam M., Alam M., Suresh Kumar R., Adil S.F. (2017). Synthesis, crystal structure and Hirschfeld surface analyses of an alkyl amine based salt, [C_5_H_16_N_2_][ZnCl_4_] and its enzyme inhibition activity. J. Saudi Chem. Soc..

[35] Olson G.B. (1987). Interphase kinematics and the roles of structure and composition in solid-state transformations. Scr. Metall..

[36] Greco K., Bogner R. (2012). Solution-mediated phase transformation: Significance during dissolution and implications for bioavailability. J. Pharm. Sci..

[37] Allan M.C., Owens B., Mauer L.J. (2020). Relative humidity-temperature transition boundaries for anhydrous β-caffeine and caffeine hydrate crystalline forms. J. Food Sci..

[38] Zhao J., Yang P., Fu J., Wang Y., Wang C., Hou Y., Shi Y., Zhang K., Zhuang W., Ying H. (2022). Polymorph control by designed ultrasound application strategy: The role of molecular self-assembly. Ultrason. Sonochem..

