# Supplementary material for: Solid Forms of Bio-Based Monomer Salts for Polyamide 512 and Their Effect on Polymer Properties
Source: Polymers (Basel). 2024 Oct 22;16(21):2953. doi: 10.3390/polym16212953 (PMC11548595; doi:10.3390/polym16212953)
Supplement: Supplementary file 1 [file polymers-16-02953-s001.zip › polymers-3214822-supplementary.pdf]

Solid forms of Bio-based Monomer Salts for Polyamide 512 and Their Effect on Polymer Properties

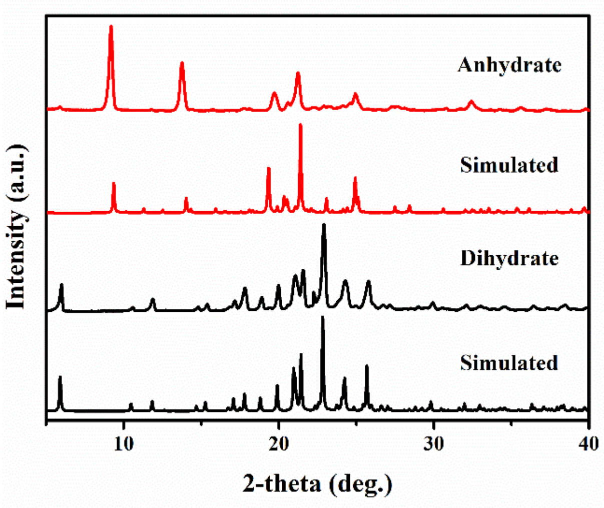

Figure. S1 Comparison of powder X-ray diffraction (PXRD) patterns with the simulated patterns from single-crystal X-ray diffraction (SCXRD) for the anhydrate and dihydrate of PDA-DCA.

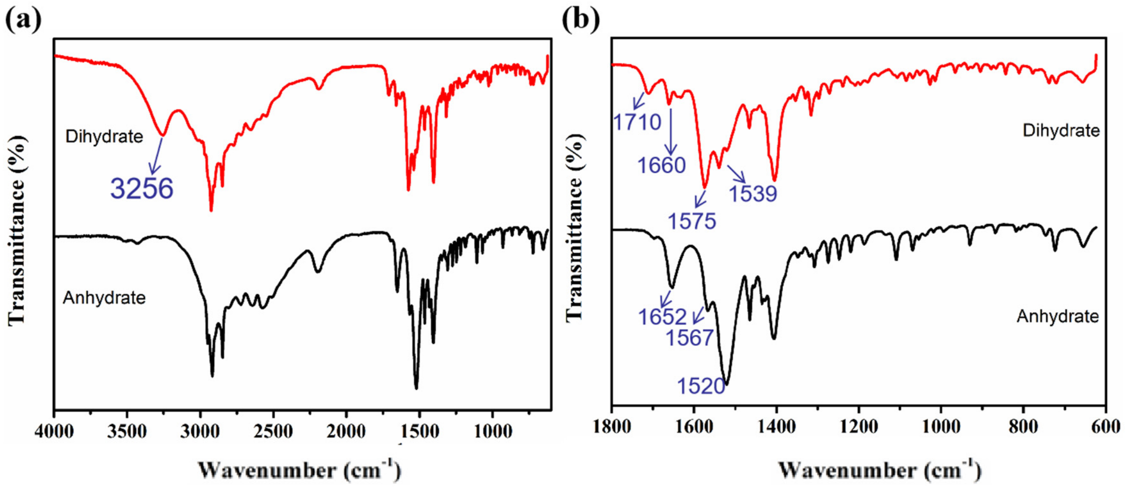

Figure. S2 Comparison of the FTIR patterns of the nylon512 salt anhydrate and dihydrate.

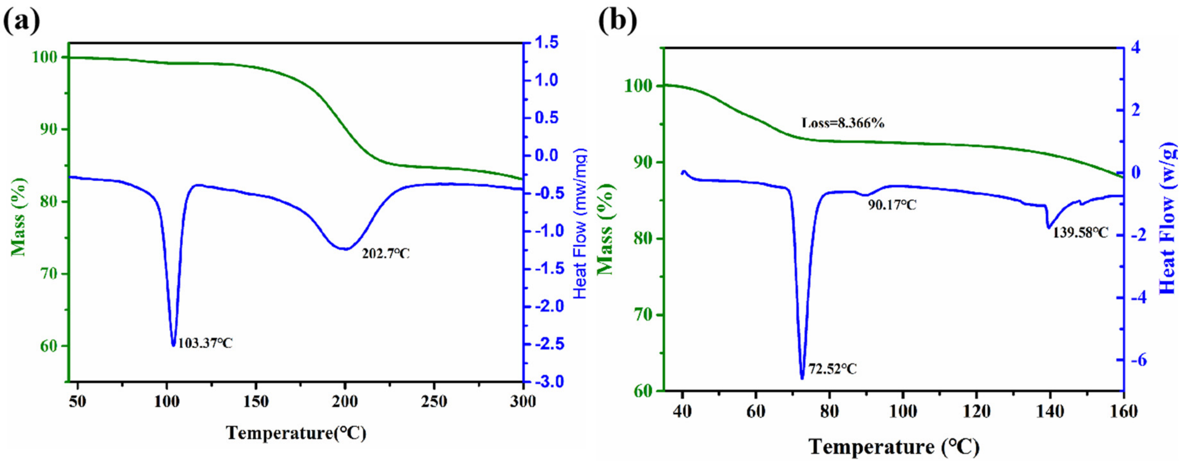

Figure. S3 DSC and TGA curves of anhydrate (a) and dihydrate (b).
